# Incidental Findings Diagnosed during Preprocedural Evaluation of TAVR

**DOI:** 10.1155/2019/7478608

**Published:** 2019-04-02

**Authors:** Levent Sahiner, Cem Coteli, Ahmet Kivrak, Yusuf Ziya Sener, Serkan Asil, Tuncay Hazirolan, Ergun Baris Kaya, Necla Ozer, Kudret Aytemir

**Affiliations:** ^1^Hacettepe University, Department of Cardiology, Ankara, Turkey; ^2^Gulhane Training and Research Hospital, Department of Cardiology, Ankara, Turkey; ^3^Hacettepe University, Department of Radiology, Ankara, Turkey

## Abstract

**Introduction:**

Transcatheter aortic valve replacement is an important therapeutic option for aortic stenosis (AS) patients who have high surgical risk. TAVR is a complex procedure. Proper preparation of the patient is of significant importance for the final success and affects the morbidity and mortality of the TAVR directly. Pre-TAVR computed tomography is one of the corner stones of these preparation steps, and many patients get some incidental diagnoses.

**Materials and Methods:**

In this trial, we have investigated 155 patients who had underwent TAVR between February 2013 and March 2017 at Hacettepe University Adult Hospital Cardiology Clinic.

**Results:**

Total number of incidental diagnoses was 541, and 451 of them were the first diagnoses. Total number of cardiovascular findings and noncardiovascular findings was 369 and 172, respectively. The most common cardiovascular finding is atherosclerotic heart disease (139, 89.6%). The most common noncardiovascular finding is pulmonary nodule (41, 26.4%). 143 of 155 patients had at least one incidental diagnosis after the reassessment, and 33 different diagnoses were identified with computed tomography. The mean STS-PROM was 8.38% (range 2.8% to 23%), and the mean STS-PROM was calculated 9.4% (range 3.6% to 23%) after the reassessment of computed tomography.

**Conclusion:**

Preprocedural evaluation is one of the most important steps in TAVR. Computed tomography imaging provides extensive information, not only for procedure planning. Our findings emphasize that computed tomography has a crucial role for the preprocedural evaluation of TAVR candidates.

## 1. Introduction

Transcatheter aortic valve replacement is an important therapeutic option for aortic stenosis (AS) patients who have high surgical risk [1]. These patients were named inoperable before the introduction of TAVR, and medical therapy was the only option. Traditionally, there were lots of suitable patients accepted as inoperable and rejected to be treated by surgery [2, 3]. Nowadays, with technical development and growing experience in this field, even moderate-risk patients are treated efficiently with TAVR [4, 5]. Together with inoperable and high-risk patients, this translates to increasing number of TAVR procedures performed worldwide [6].

It is recommended that the “heart team” of the center should decide therapeutic options for patients with severe aortic stenosis, medical, transcatheter, or surgical. There are risk scoring systems to help decision-making in these patients: “STS (the Society of Thoracic Surgeons) adult cardiac surgery risk calculator” is the most widely used. Current guidelines for the management of patients with valvular heart disease recommend using STS score for cardiac surgery risk assessment of the patients with severe AS. The guidelines recommend to classify as high risk if the score is higher than 8–10% [7, 8].

TAVR is a complex procedure. Proper preparation of the patient is of significant importance for the final success and directly affects the morbidity and mortality of the TAVR [9]. Evaluation of aortic root anatomy, coronary, and iliofemoral arteries is vital for this preparation. The results of these diagnostic work-ups are not only used for the procedure planning, selection of the access site, and determination of the heart valve size, but also give valuable information about possible complications, providing an opportunity for the operators to take precautions [10].

Traditionally, transthoracic and transesophageal echocardiography, together with invasive angiography, were used for the evaluation of aortic valve and vascular anatomy [11]. However, with the acknowledgement of efficacy of computed tomography to comprehensively address all issues in the preparative diagnostic work-up of TAVR patients, its use is now recognized as the mainstay of pre-TAVR preparation [12].

As computed tomography scans provide detailed imaging information for the patients being prepared for TAVR, many patients get some incidental diagnoses. Some of these new diagnoses could possibly change the mortality and morbidity of the procedure as well as calculated surgical risk for the patient.

In this study, we aimed at investigating the prevalence of incidental findings in pre-TAVR computed tomography imaging and understanding the influence of these incidental findings on surgical risk assessment.

## 2. Materials and Methods

In this trial, we have investigated 155 patients who presented to the Hacettepe University Department of Cardiology and were evaluated for TAVR between February 2013 and March 2017. We have reevaluated these patients with their preoperative computed tomography retrospectively. We aimed at defining their incidental diagnoses obtained in preoperative computed tomography and recalculating their cardiac surgery risk.

### 2.1. Study Population

153 patients who had undergone TAVR successfully in the Hacettepe University Department of Cardiology and 2 patients who cancelled TAVR procedure because of inappropriate vascular access and renal cell carcinoma have been included in the study.

The patients who were cancelled because of the heart team opinion, low and moderate risk status, were excluded. All the patients were evaluated by full-body computed tomography for the decision of vascular access after TAVR decision of the heart team. Informed consent was signed by all the study population.

### 2.2. Computed Tomography Scan

CT examinations were performed with a first-generation DSCT system (SOMATOM, Definition; Siemens, Forchheim, Germany) that used a standardized imaging protocol with retrospective electrocardiogram (ECG) gating for cardiac imaging and nongated scan for the aorta and pelvic arteries. The cardiac scan was located between the carina and diaphragmatic border of the heart. The imaging parameters for cardiac examination were as follows: slice collimation, 2 × 64 × 0.75 mm; gantry rotation time, 320 milliseconds; and tube voltage and current based on patient weight (100 kV/320 mAs in patients with BMI < 30, 120 kV/320 mAs in patients with BMI > 30). ECG gating dose modulation was used. The ECG window of full-tube current was set between 30% and 80%. Nongated aorta pelvic angiography was obtained 10 minutes after the cardiac scan between the thoracic inlet and femoral neck with a slice thickness of 1 mm and tube voltage of 120 kV. For contrast enhancement, iopromide (Ultravist 370; Bayer Healthcare, Germany) was used via a 16/18 gauge access in the right cubital vein by using a biphasic contrast medium injection protocol for both cardiac and aorta pelvic angiography (80 ml at a flow of 5 ml/s for cardiac scan, and 50 ml at a flow of 4 ml/s for aorta pelvic scan). Contrast medium injection was followed by a saline chaser bolus at a dose of 30 ml and flow rate of 5 and 4 ml/s, respectively.

### 2.3. Analysis of the Computed Tomography Images

The computed tomography images were analyzed by two experienced radiologists, and they have defined all cardiovascular and noncardiovascular findings. And both groups were categorized as significant and nonsignificant.

## 3. Results

### 3.1. Baseline Characteristics

The study group consisted of 155 patients (mean age 76.9, range 51 to 94 years). There were 98 women (63.2%) and 57 men (36.8%).

The number of patients who had atherosclerotic heart disease was 84 (54%), diabetes mellitus was 43 (28%), hypertension was 103 (66%), and chronic obstructive lung disease was 35 (22%). The mean STS-PROM was 8.38% (range 2.8% to 23%), and logistic EuroSCORE was 33.9% (range 18% to 65%) (Table 1).

### 3.2. Computed Tomography Findings

Total findings were 541, and 451 of them were first diagnoses. Cardiovascular findings were 369, noncardiovascular findings were 172. The most common cardiovascular finding is atherosclerotic heart disease (139, 89.6%). The most common noncardiovascular finding is pulmonary nodule (41, 26.4%). All patients had diagnoses (known or new) with computed tomography. There were 3.5 total and 2.9 incidental diagnoses per patient with CT. 142 of 155 patients had at least one new incidental diagnosis not known before the CT assessment.

### 3.3. Cardiovascular Findings

**Figure 1:**
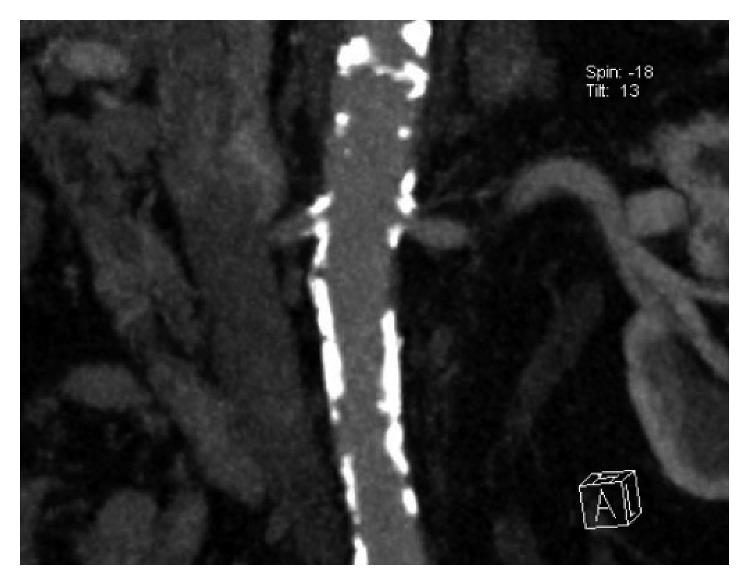
Bilateral renal artery stenosis.

The most common cardiovascular finding was atherosclerotic heart disease (*n*: 139; 89.6%). Hemodynamically significant coronary heart disease was present in 90 patients (58%). The patients who had atherosclerotic heart disease were 85 before computed tomography scan. 54 patients had new atherosclerotic heart disease diagnosis with computed tomography. The second common cardiovascular finding was peripheric artery disease (*n*: 111; 71.6%), including renal artery stenosis (Figure 1), carotid artery stenosis, descendant aortic disease, and iliac and femoral artery disease. Renal artery stenosis and carotid artery stenosis were found in 42 patients (27%) and 21 patients (13.5%), respectively. 2 patients had renal artery stenosis and carotid artery stenosis together. The enlargement of the pulmonary artery was found in the computed tomography images of 53 patients (34.1%). Ascendant aortic dilatation was found in 32 patients (20.6%), and aortic aneurism was found in 11 patients (7%). Other significant cardiovascular findings were abdominal aortic dissection in 1 patient (0.6%), pericardial effusion in 13 patients (8.3%), intracardiac thrombus in 3 patients (1.8%), and bicuspid aortic valve in 2 patients (1.2%).

Nonsignificant cardiovascular findings were unilateral duplicated renal artery, persistent left superior vena cava, and retroaortic renal artery which existed in 3 patients (1.8%), 1 patient (0.6%), and 1 patient (0.6%), respectively (Table 2).

### 3.4. Noncardiovascular Findings

Total noncardiovascular findings were 172. 118 of 172 were categorized as the significant finding, and 54 of 172 were nonsignificant.

**Figure 2:**
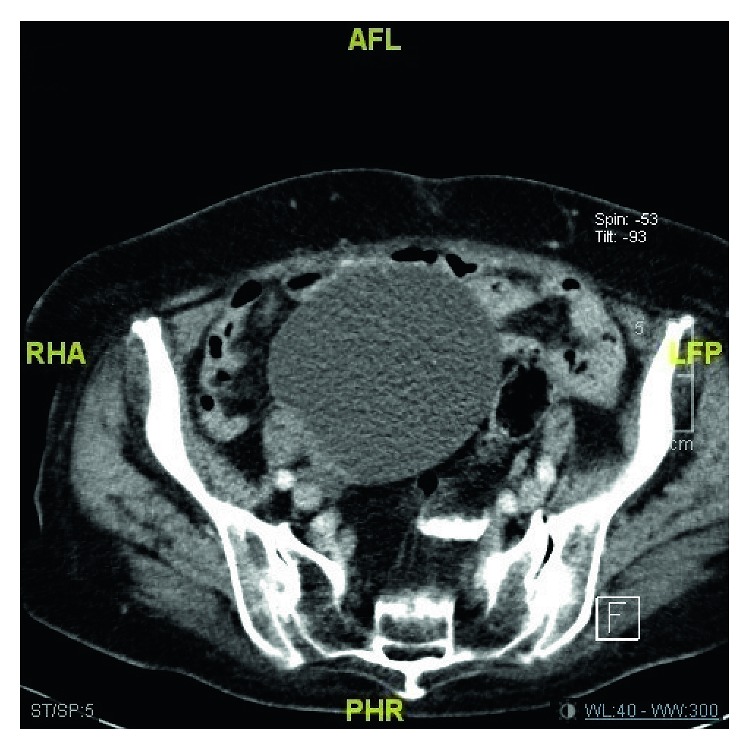
Huge adnexal mass.

The most common noncardiovascular significant finding was pulmonary nodule (*n*: 41; 26.4%). Other nonsignificant findings were pleural effusion in 34 patients (21.9%), lymphadenopathy in 30 patients (19.3%), thyroid nodule in 8 patients (5.1%), huge adnexal mass (Figure 2) in 1 patient (0.6%), and renal cell carcinoma in 1 patient (0.6%).

**Figure 3:**
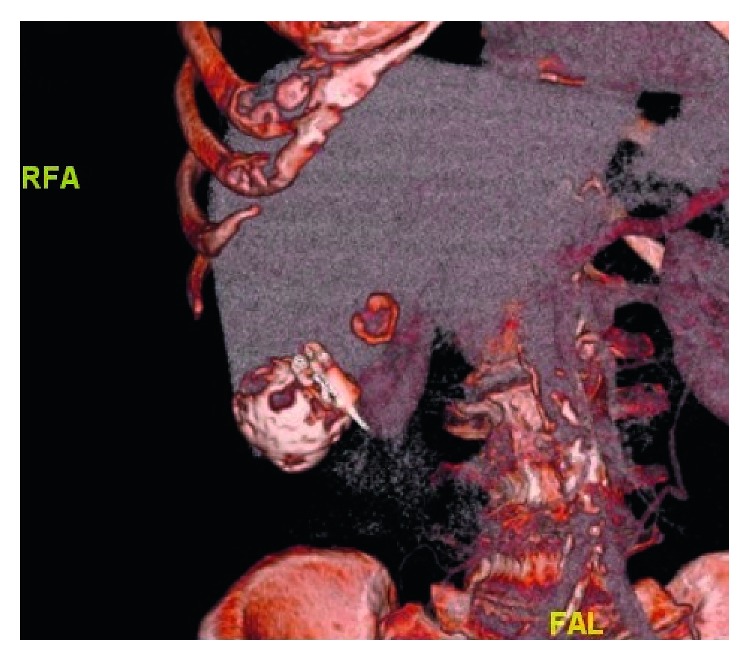
Hydatid cyst, 3D reconstructed.

The most common noncardiovascular nonsignificant finding was simple renal cyst (*n*: 29; 18.7%) (Table 3). Liver cyst (Figure 3) was observed in 6 patients (3.8%), and they were evaluated after TAVR.

### 3.5. Recalculation for the Risk Assessment

142 of 155 patients had at least one incidental diagnosis after the reassessment, and 33 different diagnoses were identified with computed tomography. Some of these diagnoses (coronary heart disease, etc.) directly affected the risk assessment score. The mean STS-PROM was 8.38% (range 2.8% to 23%), and the mean logistic EuroSCORE was 33.9% (range 18% to 65%) before reassessment. The mean STS-PROM was calculated 9.4% (range 3.6% to 23%) and the mean logistic EuroSCORE was calculated 38.3% (range 25% to 65%) after the reassessment of computed tomography.

## 4. Discussion

In our study, we have reassessed the computed tomography of the patients who had high cardiovascular surgery risk for symptomatic aortic stenosis and undergone TAVR or were evaluated for TAVR and cancelled after CT scan in Hacettepe University Cardiology Clinic. The main purpose of this study was the determination of the incidental findings in pre-TAVR computed tomography images and trying to understand the importance of these findings.

To our knowledge, aortic stenosis in elder patients is caused by sclerodegenerative process generally and shares the same risk factors with atherosclerosis [13]. As supportive information to this knowledge, the most common findings in computed tomography were atherosclerotic heart disease in our study. Most of the patients have already had atherosclerotic heart disease diagnosis before reassessment. But we should underline a considerable number of the patients (*n*: 55) had atherosclerotic heart disease diagnosed firstly with pre-TAVR computed tomography. Other papers published before were focused on noncardiac findings on computed tomography [14–16]. So those studies did not mention the relation between senile aortic stenosis and atherosclerotic heart disease. However, diagnosis of incidental atherosclerotic heart disease is important to show this relation and emphasizes the same pathophysiologic origin. Our study is the first for the reassessment of the pre-TAVR computed tomography for all incidental diagnoses and underlines the relation between sclerotic AS and atherosclerotic cardiovascular disease.

Peripheric artery disease (*n*: 111; 71.6) is another common finding in our series. We underline that there were only 7 patients who had peripheral artery disease diagnosis already. We think that asymptomatic peripheric artery disease is the main reason of this high incidental diagnosis amount. Second reason might be the masked symptoms because of decreased exertional capacity of severe aortic stenosis patients. On the contrary, we should emphasize that 3 of our patients had undergone TAVR procedure with transsubclavien approach because of inappropriate femoral access, and 1 of our patients was cancelled because there was not any appropriate vascular access. And none of these 4 patients had peripheral artery disease before pre-TAVR CT.

In our study, the most common noncardiovascular finding was pulmonary nodule. This result is the same with the Hussien et al. paper [15]. Hussien et al. told that there were 59 (28.2%) patients in 209 patients who were going to TAVR. The incidence of the pulmonary nodule is similar in our series (41/154 (26.6%)). In our study, other noncardiovascular findings thyroid nodule, adrenal adenoma, adnexal mass, gallbladder wall thickness are categorized as the significant finding. The reason for this classification is this incidental adenomas and masses could be the images of any malignancy and could affect the morbidity and mortality of the patients directly, independent from the operation of percutaneous valve implantation. As we know, STS or EuroSCORE calculation does not include any scores for malignancy. Classically, TAVR is accepted as an inappropriate therapy for the patients who have a life expectancy lower than one year [4, 5]. According to this information, we propose that the patients should be examined after computed tomography again to determine the suitability for TAVR, especially to determine the influence of the incidental findings on patient life expectancy.

All patients who had significant nonvascular findings were consulted with chest disease, endocrinology, urology, and gynecology departments. The decision of all patients, except adnexal mass and renal cell carcinoma, was follow-up. The suggestion of gynecology department for the patient who had adnexal mass was surgery. But severe aortic stenosis created elevated risk for the patient. So, this patient was reevaluated by the heart team and decided to perform TAVR then adnexal surgery to reduce cardiovascular preoperative risk. The patient who had diagnosed renal cell carcinoma was consulted to oncology, and the patient was acknowledged as inoperable and did not have more than one-year survey. So, TAVR procedure was cancelled for this patient.

We classified the pleural effusion in 34 patients and lymphadenopathy in 30 patients as a significant finding also. Physicians should not accept all pleural effusions as a result of heart failure and lymphadenopathy as a result of a simple infection. We should not forget these findings could be results of any malignancy as well, especially in this TAVR age group. Incidental findings should be evaluated carefully together with symptoms and signs of the patient, to rule out or diagnose a significant disorder like malignancy. Any diagnosed significant disorder may change the way of therapy, or make TAVR futile for this patient.

In our study, we should emphasize the change of STS-PROM and logistic EuroSCORE after the reassessment of the computed tomography. The mean STS-PROM is 8.38% (range 2.8% to 23%) before reassessment and 9.4% (range 3.6% to 23%) after reassessment in our series. Atherosclerotic heart disease and peripheral artery disease may change these scores and may change the opinion on patients about the surgical risk status. So, we offer that computed tomography findings should not be forgotten while assessing the risk, and if it is necessary, physicians should recalculate these scores with new findings and reevaluate with the heart team.

At last, our study has some limitations. First of all, this is a retrospective designed study. Secondly, data of the study are from a single center and single country. Population of study is relatively homogenous, and these results could show some variety in different population. Finally, the study population is relatively small. In large scale, similar studies can have more power to show infrequent incidental diagnosis. Despite these limitations, our results are important for emphasizing frequency of incidental diagnosis and importance of preprocedural evaluation of TAVR procedure.

## 5. Conclusion

Preprocedural evaluation is one of the most important steps in TAVR. Computed tomography imaging provides extensive information not only for procedure planning, access site selection, and valve sizing, but also for new cardiovascular or noncardiovascular diagnosis which could potentially change the management of the patient. Our findings emphasize the crucial role of computed tomography for the preprocedural evaluation of TAVR candidates and support its widespread use in this context.

## Figures and Tables

**Table 1:** Baseline characteristics.

	Patients (*n*)
Age	76.9 years (range 51 to 94 years)
Sex	
Women	98 (63.6%)
Men	56 (36.4%)
Coronary heart disease	84 (54%)
Hypertension	103 (66.6%)
Diabetes mellitus	43 (28%)
Chronic obstructive lung disease	35 (22.2%)
Chronic kidney disease	62 (40%)
Cerebrovascular event	18 (11%)
Peripheric artery disease	7 (4%)
Atrial fibrillation	34 (22%)
Ejection fraction	49% (range 15% to 74%)
Mean aortic valve area^*∗*^	0.74 (range 0.4 to 1.0)
MVR	8 (1%)
Bioprosthesis	2
Metallic	6

^*∗*^Excluded the patients with severe aortic regurgitation

**Table 2:** Cardiovascular findings in computed tomography.

	Patients (*n*)
*Significant findings*	
Atherosclerotic heart disease	139 (89.6%)
(i) Coronary artery disease (critical stenosis)	90 (58%)
Peripheral artery disease	111 (71.6%)
(i) Rather than renal artery and carotid artery stenosis	50 (32%)
(ii) Renal artery stenosis	42 (27%)
(iii) Carotid artery stenosis	21 (13.5%)
Increased pulmonary artery diameter	53 (34%)
Ascending aorta dilatation	32 (20.6%)
Pericardial effusion	13 (8.3%)
Aortic aneurysm	11 (7%)
Intracardiac thrombus	3 (1.8%)
Bicuspid aorta	2 (1.2%)
Aortic dissection	1 (0.6%)

*Nonsignificant findings*	
Double unilateral renal artery	3 (1.8%)
Retroaortic renal artery	1 (0.6%)
Persistent left superior vena cava	1 (0.6%)

**Table 3:** Noncardiovascular findings in computed tomography.

	Patients (*n*)
*Significant Findings*	
Pulmonary nodule	41 (26.4%)
Pleural effusion	34 (22%)
Lymphadenopathy	30 (19.3%)
Thyroid nodule	8 (5.1%)
Renal cell carcinoma	1 (0.6%)
Adrenal adenoma	1 (0.6%)
Adnexal Mass	1 (0.6%)
Gallbladder wall thickening	1 (0.6%)
Glomus tumor	1 (0.6%)

*Nonsignificant Findings*	
Renal cyst	29 (18.7%)
Hiatal hernia	7 (4.2%)
Liver cyst	6 (3.8%)
Cholelithiasis	5 (3.2%)
Nephrolithiasis	2 (1.2%)
Accessory kidney	1 (0.6%)
Baker cyst	1 (0.6%)
Thoracic outlet syndrome	1 (0.6%)
Pleural lipoma	1 (0.6%)
Inguinal hernia	1 (0.6%)

## Data Availability

The data used to support the findings of this study are available from the corresponding author upon request.
